# Splenic infarction in babesiosis: A rare presentation

**DOI:** 10.1002/ccr3.2301

**Published:** 2019-07-11

**Authors:** Ashish Gupta, Poras Patel, Kapil Manvar, Timothy Kellner, Elizabeth Guevara

**Affiliations:** ^1^ Internal Medicine The Brooklyn Hospital Center New York NY USA; ^2^ Hematology Oncology The Brooklyn Hospital Center New York NY USA

**Keywords:** babesiosis, hemolytic anemia, peripheral smear, splenic infarction

## Abstract

Babesiosis is a protozoan parasitic infection transmitted by the *Ixodes* tick. Splenic infarction is a rare, but potentially life‐threatening complication of babesiosis; it is therefore vital that this complication is recognized.

## 1 BACKGROUND

Splenic infarction is a rare complication of babesiosis, and few reports exist. A 53‐year‐old man presented with flu‐like symptoms, fever, abdominal pain, and a syncopal episode after a recent hike. Investigations revealed hemolytic anemia, thrombocytopenia, and multiple splenic infarcts. He was diagnosed with babesiosis and improved with therapy.

Babesiosis is a protozoan parasitic infection that is most commonly caused in humans by the *Babesia microti* (*B microti*) parasitic strain. It is transmitted by *Ixodes* ticks and is endemic in the Northeastern and Upper Midwestern United States, particularly in New England, New York, New Jersey, Wisconsin, and Minnesota.^1^ Presentation ranges from asymptomatic or a mild flu‐like illness to severe fatal disease, the latter of which is particularly prevalent in immunocompromised patients. Diagnosis is generally made by classic findings on a peripheral blood smear.

Splenic infarction is a rare complication in patients with babesiosis and has been described in few cases, even in patients with the less severe form of the disease. Here, we present a case of babesiosis with infarction of the spleen in an Asian male.

## 2 CASE HISTORY

A 53‐year‐old Asian male presented to our hospital with fever, chills, left upper quadrant pain, and generalized weakness of 1‐week duration. His symptoms progressively worsened and also experienced an episode of syncope. Two weeks prior to his symptoms, he embarked on a hiking trip to the Harriman State Park in New York. Review of systems was negative for rash, vomiting, diarrhea, joint pain, shortness of breath, cough, facial palsy, sick contacts, animal contact, melena, hematochezia, and hematuria. The patient was otherwise healthy, had no significant medical history, and was not taking any medications. His surgical history included a cholecystectomy performed 20 years ago. He was a chronic smoker, but denied use of alcohol and illicit drugs.

## 3 INVESTIGATIONS AND TREATMENT

**Figure 1:**
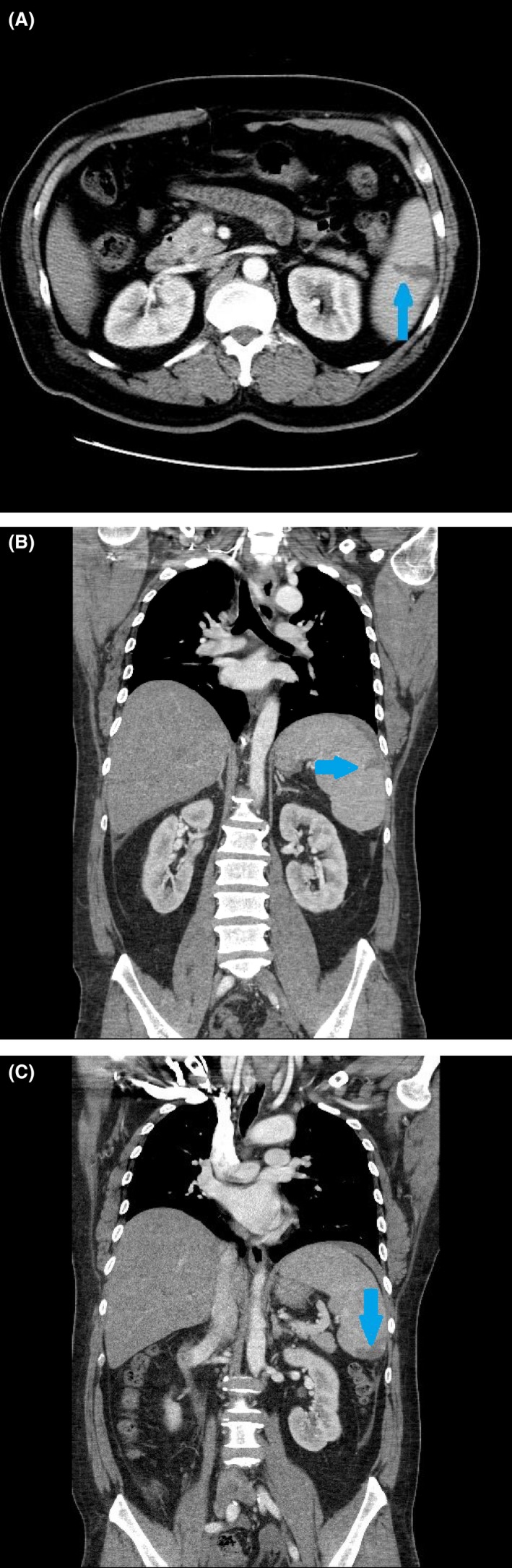
Contrast‐enhanced computed tomography of the abdomen. Coronal (A, B) and axial (C) contrast‐enhanced computed tomography scans of the abdomen demonstrating several peripheral hypodense wedge‐shaped lesions compatible with splenic infarction

**Figure 2:**
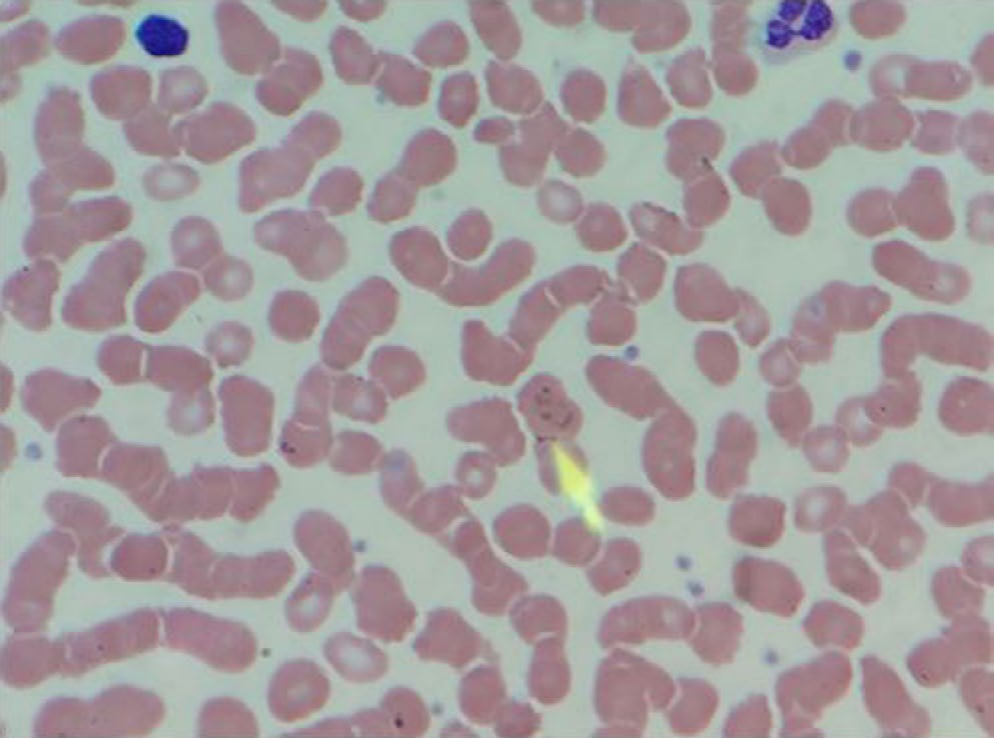
Peripheral blood smear. A peripheral blood smear with an arrow illustrating the Babesia parasite within RBCs

The physical examination was significant for a temperature of 38.8°C, conjunctival pallor, jaundice, and left upper quadrant tenderness. The initial laboratory results revealed a hemoglobin concentration of 11.2 g/dL, leukocyte count of 5300 cells/mm^3^, platelet count of 90 000/mm^3^, aspartate aminotransferase level of 38 U/L, alanine aminotransferase level of 62 U/L, alkaline phosphatase level of 270 U/L, total bilirubin of 2.6 mg/dL, indirect bilirubin of 1.4 mg/dL, lactate dehydrogenase level of 279 U/L, and haptoglobin of <8 mg/dL. A computed tomography (CT) scan of the abdomen revealed multiple wedge‐shaped infarcts within the spleen and a small amount of perisplenic fluid (Figure 1). The peripheral blood smear revealed many ring forms of parasites within the red blood cells (RBCs), with approximately 1.5% parasitemia (Figure 2). Many inclusion bodies were noted within the leukocytes, and many platelet clumps were also observed.

A primary care physician diagnosed patient with babesiosis about four days before hospitalization. The patient was started on treatment with azithromycin and atovaquone, but remained febrile and experienced little improvement in his other symptoms.

Treatment was continued with azithromycin (500 mg once then 250 mg every 24 hours) and atovaquone (750 mg every 12 hours). Doxycycline (100 mg every 12 hours) was added empirically to the patient's antimicrobial regimen for potential tick‐borne co‐infection with *Borrelia* or *Anaplasma* species with pending confirmatory studies. Doxycycline was later discontinued after negative serological tests.

## 4 OUTCOME

The patient defervesced within 48 hours of hospitalization with significant clinical improvement. However, he remained anemic and his transaminase and lactate dehydrogenase levels were slow to improve. The patient was discharged on day 4 after hospitalization with a hemoglobin level of 10.4 g/dL and a hematocrit of 31% with normal indices.

## 5 DISCUSSION

Human babesiosis is a zoonotic infection caused by intraerythrocytic protozoa of the genus *Babesia*. Babesiosis was first reported in 1888 by Victor Babes as the cause of hemoglobinuria in cattle,^2^ and by the 1950s, cases in humans were reported. The first case in an immunocompetent individual was reported in 1969.^3^ Multiple *Babesia* species are known to cause disease in humans, with *B microti* and *Babesia divergens* being the most common etiologies in North America and Europe, respectively.^4^, ^5^, ^6^, ^7^ *Babesia microti* is endemic in the Northeastern and Upper Midwestern United States, which can be largely attributed to the ecological distribution of its tick vector, *Ixodes scapularis*. This is also the primary vector of *Borrelia burgdorferi* and *Anaplasma phagocytophilum*, which cause Lyme disease and Human Granulocytic Anaplasmosis, respectively.^8^, ^9^, ^10^, ^11^, ^12^ Multiple studies have identified the white‐footed mouse (*Peromyscus leucopus*) as the principal host of *B microti*.^9^, ^10^, ^13^

Although, like that of our patient, vector transmission of *B microti* is the most common cause of babesiosis in humans, particularly in endemic areas such as the Northeastern United States, other modes of transmission have been described. Patients receiving blood transfusions are at risk, as *B microti* is the most common transfusion‐transmitted parasite,^14^ and due to the intraerythrocytic life cycle of the parasite, leukoreduction of blood products does not decrease transmission.^6^, ^14^ The typically asymptomatic course of infection in immunocompetent individuals, as well as a lack of routine serological tests, may contribute to the majority of unreported cases. *Babesia microti* infection during pregnancy can also result in transplacental transmission and subsequent neonatal infection.^15^

Presentation of babesiosis can range from no symptoms^16^ to severe and even fatal disease. Immunocompetent individuals are typically asymptomatic or experience mild illness. Symptoms develop 1‐6 weeks following inoculation, and in mild disease, these may resemble a flu‐like illness, including fever, headache, chills, sweats, myalgias, and malaise. Less common symptoms associated with mild disease include anorexia, depression, headache, neck stiffness, nonproductive cough, vomiting, and dark urine. On physical examination, hepatosplenomegaly, jaundice, and retinal infarction may be detected.^5^, ^13^, ^17^ Our patient demonstrated a number of these constitutional symptoms along with left upper quadrant abdominal tenderness and a syncopal episode. Acute, severe babesiosis is typically observed in patients who are immunocompromised,^18^, ^19^ aged >50 years,^20^ or asplenic.^21^, ^22^ These patients are prone to significant complications, such as adult respiratory distress syndrome; disseminated intravascular coagulation; congestive heart failure; renal failure; pulmonary edema; persistent, relapsing disease; retinal infarct; and splenic infarct or rupture.^5^, ^17^, ^23^ Co‐infection with *B burgdorferi* or *A phagocytophilum* has also been demonstrated to increase the severity and duration of disease.^4^, ^11^, ^12^, ^24^, ^25^ Meldrum et al^26^ reported a 5% mortality rate in a retrospective study of 136 cases of *B microti* infections in New York.

Diagnosis of babesiosis begins with a thorough history taking entailing patient travel to endemic areas, recent tick exposure, blood transfusions, and history of splenectomy. Our patient reported a recent hike in New York, which is a significant risk factor. Positive lab findings are largely due to hemolytic anemia, resulting in normochromic, normocytic anemia, low hemoglobin, hematocrit, and haptoglobin, and elevated lactate dehydrogenase, total bilirubin, and reticulocyte count.^5^, ^17^ Thrombocytopenia is also a common laboratory manifestation^5^, ^17^, ^27^, ^28^, ^29^, ^30^ and may occur due to several mechanisms including decreased production, hypersplenism, widespread consumption from diffuse endothelial damage, and immune‐mediated destruction.^31^ In some cases, hepatic dysfunction can be characterized by mild elevation of aspartate/alanine aminotransferase, elevated alkaline phosphatase, and hyperbilirubinemia.^4^, ^27^, ^32^ These findings are consistent with those observed in our patient. Definitive diagnosis can be made with Wright or Giemsa‐stained thin peripheral blood smears. Often during acute infection, signet rings are detected within erythrocytes. However, this finding is not specific for *Babesia*, as the rings resemble those associated with intraerythrocytic *Plasmodium* trophozoites. A rare pathognomonic finding on peripheral blood smear is the occurrence of a Maltese cross (tetrads of immature merozoites).^4^, ^5^, ^6^, ^13^ Parasitemia can also be determined from peripheral blood smears, with levels rarely exceeding 5% in immunocompetent individuals but increasing to ≤85% in severe disease.^4^ Our patient had parasitemia of 1.5%, which correlated with his mild symptoms and immunocompetent status. Polymerase‐chain reaction testing is more sensitive than peripheral blood smear and may be used in cases where the diagnosis is indeterminate.^33^ Serology via an indirect immunofluorescence assay for *Babesia*‐specific immunoglobulin (Ig)M and/or IgG antibodies may also be used to support the diagnosis.^4^, ^5^, ^13^

Traditionally, treatment for babesiosis consists of a 7‐ to 10‐day course of quinine and clindamycin. However, treatment with atovaquone and azithromycin has been demonstrated to be equally effective and associated with fewer adverse reactions. Krause et al^23^ performed a randomized trial of the two regimens in patients with non–life‐threatening babesiosis. Adverse reactions were reported in 15% of those treated with azithromycin and atovaquone, and in 72% of those treated with quinine and clindamycin. Conversely, in severe disease, quinine and clindamycin are utilized due to insufficient data to support the use of atovaquone‐azithromycin. Wormser et al^34^ have also reported cases of atovaquone‐azithromycin resistance in severely immunocompromised patients, further limiting this regimen's utility in severe disease. Our patient was prescribed an atovaquone‐azithromycin regimen with doxycycline therapy to provide empiric coverage for co‐infection with *Borrelia* and/or *Anaplasma* species. In patients with high‐grade parasitemia (>10%), severe anemia (<10 g/dL), or significant complications, red blood cell exchange transfusion should be considered in addition to drug therapy.^21^, ^35^, ^36^, ^37^, ^38^

Splenic infarction is a rare complication in babesiosis that has been reported in very few (babesiosis) cases.^27^, ^32^, ^39^ Although our patient's age (>50 years) is a risk factor for severe disease, this case reveals that significant complications from babesiosis may arise in the setting of immunocompetent individuals with low levels of parasitemia. In all reported cases, CT scans of the abdomen without contrast revealed hypodense lesions within the patient's spleen. The mechanism of splenic infarction remains ambiguous, with one study by Wozniak et al^40^ demonstrating microthrombi formation in the smaller vessels of the spleen of hamsters inoculated with *Babesia*, resulting in coagulative necrosis. In addition, another mechanism of splenic infarct in babesiosis is reported to be similar to that in malaria wherein the parasitized RBCs are unable to deform and thus cause obstruction and then infarction of the affected area. Splenic rupture has also been reported in very few cases,^28^, ^29^, ^30^ with Froberg et al^28^ indicating that this may be due to a combination of excessive splenic histiocyte phagocytosis of infected erythrocytes and sequestration of platelets by the spleen.

## 6 CONCLUSIONS

This report describes a rare case of babesiosis with infarction of the spleen. This case highlights the importance of obtaining detailed history; considering babesiosis as a differential diagnosis in hemolytic anemia, particularly in endemic areas such as New York; accurate diagnosis from the peripheral smear; and the consideration of a rare, potentially life‐threatening complication such as splenic infarct and further possibly splenic rupture in such immunocompetent patients.

## CONFLICT OF INTEREST

None declared.

## AUTHOR CONTRIBUTIONS

AG: was involved in manuscript preparation, background research, drafting of the manuscript, and critical editing of the manuscript. PP: was involved in manuscript preparation, background research, drafting of the manuscript, and critical editing of the manuscript. KM: was involved in background research and drafting of the manuscript. TK: was involved in background research and drafting of the manuscript. All the authors approve the submitted manuscript.
